# Appropriate noise addition to metaheuristic algorithms can enhance their performance

**DOI:** 10.1038/s41598-023-29618-5

**Published:** 2023-03-31

**Authors:** Kwok Pui Choi, Enzio Hai Hong Kam, Xin T. Tong, Weng Kee Wong

**Affiliations:** 1 Department of Statistics and Data Science, National University of Singapore, Singapore, 117546 Singapore; 2 Department of Computer Science, National University of Singapore, Singapore, 117417 Singapore; 3 Department of Mathematics, National University of Singapore, Singapore, 119076 Singapore; 4 Department of Biostatistics, University of California at Los Angeles, Los Angeles, 90095 USA

**Keywords:** Engineering, Mathematics and computing

## Abstract

Nature-inspired swarm-based algorithms are increasingly applied to tackle high-dimensional and complex optimization problems across disciplines. They are general purpose optimization algorithms, easy to implement and assumption-free. Some common drawbacks of these algorithms are their premature convergence and the solution found may not be a global optimum. We propose a general, simple and effective strategy, called heterogeneous Perturbation–Projection (HPP), to enhance an algorithm’s exploration capability so that our sufficient convergence conditions are guaranteed to hold and the algorithm converges almost surely to a global optimum. In summary, HPP applies stochastic perturbation on half of the swarm agents and then project all agents onto the set of feasible solutions. We illustrate this approach using three widely used nature-inspired swarm-based optimization algorithms: particle swarm optimization (PSO), bat algorithm (BAT) and Ant Colony Optimization for continuous domains (ACO). Extensive numerical experiments show that the three algorithms with the HPP strategy outperform the original versions with 60–80% the times with significant margins.

## Introduction

Over the past couple of decades, Swarm Intelligence has and continues to inspire a steadily rising numbers of nature-inspired swarm-based algorithms for optimizing high-dimensional complex cost functions, including those that do not have analytic forms. Examples of swarm-based algorithms include particle swarm optimization (PSO), bat algorithm (BAT), ant colony optimization for continuous optimization (ACO). Swarm-based optimization algorithms are motivated by nature or animal behavior and then thoughtfully formulated into an algorithm that iterates to the optimum based on a couple of equations. Generally, these algorithms are easy to code and implement, and do not need gradient information or technical assumptions for them to generally work well. Computer codes are widely and freely available, which have undoubtedly fueled numerous and various applications of these algorithms to tackle many different types of complex real-world optimization problems. Documentation of their effectiveness is widespread and their meteoric rise in applications and interest in both industry and academia is well documented, see^1,2^, for example. Applications of these algorithms are diverse and include estimating parameters in mixed nonlinear pharmacokinetic and pharmacodynamic models^3^ and tackling various optimization problems in energy conservation^4^, medical sciences^5^ and agriculture^6^. A most recent review of PSO applications in different areas is^7^.

There are known challeneges of nature-inspired swarm-based optimization algorithms, and we highlight two: (1) They require effective tuning of their parameters to achieve optimal performance^8^; and (2) they often suffer from premature convergence to a local optimum of the cost function, especially when the cost function is high-dimensional and multi-modal^9,10^. In recent years, Yang et al. and Choi et al.^11–13^ proposed general strategies to tackle the first challenege using different tools. We opine that tuning parameters is less of an issue now with the introduction of the racing algorithm^14,15^.

In this work, we propose an innovative and simple Perturbation–Projection (PP) strategy to address the latter challenge by adding noise to a nature-inspired swarm-based algorithm. An interesting part of the strategy is to have only half of the swarm more actively engaged in the exploration for the optimum. The proposed methodology is general, and as long as certain unrestrictive technical conditions are met, our techniques ensure almost sure convergence to a global optimum. We apply the methodology to three popular swarm-based algorithms and results from an extensive simulation not only support they tend to converge to the global optimum but additionally, show that they tend to outperform the original algorithms. In the literature, there are frequently different modifications to enhance performance of the original metaheuristic algorithm. Some seek to tackle specialized problems better and others aim to speed up the algorithm. A common aim is to avoid premature convergence and there are numerous proposals to address this issue for swarm-based algorithms; see, for example^16–19^. These methods tend to be valid either for only one specific algorithm and not for a class of algorithms. Another commonality is that they do not generally have a rigorous mathematical theory to support their improved convergence to the global optimum. The distinctive features of our proposed modifications are that they are supported by mathematical theory, simple to implement and applicable to a broad class of swarm-based algorithms.

“Stochastic enhancement of an algorithm’s exploration” section describes how we add noise to a nature-inspired metaheuristic algorithm and modify the algorithm using a Perturbation-Projection (PP) strategy, In “Applications” section, inspired by the fact that agents in many swarms are often divided to specialize in different aspects of optimization (exploration and exploitation), we introduce an additional feature, heterogeneity, in the PP strategy. We then demonstrate how to modify the original PSO, BAT and ACO algorithms, by applying the PP strategy on half of the swarm agents, to meet the required conditions for almost sure convergence to the global optimum. We denote these modified algorithms by “hm” (heterogeneously modified ) in front of their names, i.e., hmPSO, hmBAT and hmACO. In “Numerical experiments” section, we conduct extensive numerical experiments using many commonly used test functions to evaluate the usefulness of the modified algorithms. Results show that the modified algorithms are either competitive or they outperform the original algorithms. We also conduct tuning parameter analysis for the Heterogeneous Perturbation-Projection (HPP) strategies. “Conclusions” section concludes with some directions for future work.

The paper has two sets of Supplementary Material: Supplementary Material A lists the 28 test functions of various types in tabular form we used to evaluate and compare performances of the various algorithms. Supplementary Material B lists five additional test functions from CEC2017 Competition and Special Session on Constrained Single Objective Real-Parameter Optimization in Supplementary Table 2 for our numerical experiment 2, including Supplementary Figures 1 and 2 to show the relative performance of the various algorithms in different ways. Supplementary Tables 3–5 compare, respectively, the performance of PSO, BAT, ACO relative to their hm versions using test functions in Supplementary Table 2.

## Stochastic enhancement of an algorithm’s exploration

In this section, we introduce a simple and effective strategy to enhance a nature-inspired swarm-based algorithm so that the enhanced algorithm is guaranteed to converge almost surely to a global optimum. We assume the cost function can be high-dimensional, non-differentiable, non-separable or non-convex with multiple local or global optima. The key idea in the proposed algorithms is simple: we incorporate an additional stochastic component with an appropriate noise level to a swarm-based algorithm to enable it to escape from a local minimum and iterate itself to a global optiumum.

The idea of adding noise to an algorithm to improve the search process is not entirely new. Several optimization subfields have incorporated such a strategy in the search of a global optimum and they have mixed results. For example, Ding and Tan, Sun et al., Neelakantan et al., Selman et al., Chen et al., Zhou et al., Jin et al.^20–26^ considered different types of optimization problems and explored adding different types of noise and its amount to different types of search procedures to find an optimum. They include adding noise to extricate a search from a saddle point or improve local searches to applications in training neural networks or adding gradient noise to improve learning for very deep networks. Interestingly, the results are mixed, implying that introducing noise to a search process is not always advantageous.

There is also the perennial problem of searching for the optimum within the user-specified region or/and the optimum has to satisfy various constraints. There are various techniques specially designed for meeting such requirements and sample papers that address this issue in different ways are^27–31^. For example, Maurice^30^ advocated the usage of a reflecting wall to bounce back particles that went beyond the search space, and^31^ took advantage of the fact that *D*-optimal designs frequently have support points at the boundary of the search space, and suggested bringing back out-of-region search particles to the boundary. Our proposed algorithm described below has the distinguishing features that (i) the search is always confined to the user-specified region and the solution satisfies all the constraints, (ii) the strategy is quite general and applies to a wide class of swarm-based metaheuristic algorithms, and (iii) the algorithm is guaranteed to converge almost surely to a global optimum. Additionally, our work appears to be the first in the literature to add noise to a nature-inspired metaheuristic algorithm and unlike the above cited work, our results suggest that such a strategy is generally helpful for a swarm-based algorithm for finding the global optimum.

Without loss of generality, we may assume that we have a minimization problem since $\min _{{\textbf{x}}\in {\mathcal {D}}} f({\textbf{x}}) = - \max _{{\textbf{x}}\in {\mathcal {D}}}\{- f({\textbf{x}})\}$. Here $f({\textbf{x}})$ is a user-specified real-valued cost function defined on a given compact subset ${\mathcal {D}}$ of ${\mathbb {R}}^d$. For many real-world applications, there are physical and budgetary constraints and we assume that these constraints have been appropriately incorporated and formulated as a general minization problem. For many other problems, like those in machine learning, regularization terms, sometimes denoted by $\rho \Vert {\textbf{x}}\Vert _1$ (or $\rho \Vert {\textbf{x}}\Vert ^2_2$) are often added to the nonnegative loss/cost function *f* to prevent over-fitting. The regularization parameter $\rho$ can often be selected through various cross validation methods^32,33^. For such situations, the goal is then to equivalently minimize the function $F({\textbf{x}})=f({\textbf{x}})+\rho \Vert {\textbf{x}}\Vert _1$ (or $f({\textbf{x}})+\rho \Vert {\textbf{x}}\Vert ^2_2$) over ${\mathcal {D}}$, which is the closed $L_1$-ball (or $L_2$-ball) centered at the origin with radius $f({\textbf{0}})/\rho$. This is because for ${\textbf{x}}\notin {\mathcal {D}}$, $F({\textbf{x}})>F({\textbf{0}})$ so the global minima are in ${\mathcal {D}}$.

To solve the optimization problem, we resort to nature-inspired swarm-based algorithms, which are increasingly used to solve complex and high-dimensional optimization problems. They generally employ an evolutionary-like or swarm-based strategy to search for an optimum by first randomly generating a set of candidate solutions of given size *n*. Depending on the particular swarm-based algorithm, candidate solutions are called agents or particles. These algorithms have tuning parameters, embrace common search principles and have good exploration and exploitation abilities. They also include stochastic components and tuning parameters and a swarm-based type of algorithm may be generally described as follows:

At time or iteration $t=1, 2, \ldots$, let ${\textbf{x}}_i(t)$ denote the *i*th particle’s position or candidate solution of the optimization problem. Let $\{{\textbf{x}}_i(t): 1 \le i \le n\}$ denote the collection of candidate solutions of the swarm of size *n*. In addition, there is auxiliary information from each member of the swarm and we denote them collectively by $\{{\textbf{v}}_i(t): 1 \le i \le n\}$. For example, in PSO and BAT, the auxiliary information of each particle is its velocity with which it flies to the current position. We denote them by

1 $$\begin{aligned} {\textbf{x}}(t)=({\textbf{x}}_1(t),\ldots , {\textbf{x}}_n(t))~\text {and}~ {\textbf{v}}(t)=({\textbf{v}}_1(t),\ldots , {\textbf{v}}_n(t)). \end{aligned}$$

ACO does not have velocity in its formulation. Suppose the algorithm has a stochastic update rule of the following form: for agent *i*,

2 $$\begin{aligned} {\textbf{x}}_i(t+1)=\Phi _i({\textbf{x}}(t),{\textbf{v}}(t),f), \quad {\textbf{v}}_i(t+1)=\Psi _i({\textbf{x}}(t),{\textbf{v}}(t),f), \end{aligned}$$

for $1 \le i \le n$, where $\Phi _i, \Psi _i$ are some functions of which the outputs can be random. At either a pre-selected deterministic time or a stopping time *T*, the algorithm is terminated and the best solution $\min _{1 \le i \le n, 0\le t\le T} f({\textbf{x}}_i(t))$ is output.

A key desirable property of a global optimization algorithm is its ability to identify a global optimum eventually. We formulate the following conditions on the algorithm’s agents, ${\textbf{x}}_1(t),\ldots , {\textbf{x}}_n(t)$.

1. At any iteration, all agents stay in ${\mathcal {D}}$, i.e., ${\textbf{x}}_i(t) \in {\mathcal {D}}$ for $1 \le i \le n$ and $t= 0, 1, \ldots , T$.
2. Some of the agents are explorative, so they can find any regions of improvement with positive probability. Specifically, there exists an $\alpha >0$, independent of *t*, such that for any *d*-dimensional ball *B* in ${\mathcal {D}}$ satisfying $f({\textbf{y}}) \le \min _{1\le i\le n} f({\textbf{x}}_i(t)),$ for all ${\textbf{y}}\in B$, the following holds
  3 $$\begin{aligned} \max _{1 \le i \le n} {\mathbb {P}}_t( {\textbf{x}}_i(t+1) \in B ) \ge \alpha |B|, \end{aligned}$$
  with $|B|$ denoting the volume of *B* and ${\mathbb {P}}_t$ the conditional probability with information at the *t*-th iteration. This condition can often be achieved by giving a lower bound of the conidtional density. See Proposition 2 as an example.
3. The algorithm is time improving, i.e., if we let $m(t)=\min _{1 \le i \le n} f({\textbf{x}}_i(t))$, $m(t+1)\le m(t)$ almost surely. Note that (C3) itself does not guarantee the algorithm converge, since an algorithm in stalemate also satisfies it.

Now we are ready to state our first result.

### Theorem 1

Suppose *f* is a continuous function defined on a given compact subset ${\mathcal {D}}$ of ${\mathbb {R}}^d$ with at least one minimizer in the interior of ${\mathcal {D}}$. Any swarm-based algorithm that satisfies *(C1)*–*(C3)* will converge to the global minimum almost surely (a.s). In other words, if the swarm-based algorithm has *n* agents, $\lim _{t\rightarrow \infty } m(t) = \min _{{\textbf{x}}\in {\mathcal {D}}} f({\textbf{x}})$ almost surely

Moreover, for any error threshold $\epsilon >0$, there is a constant $1>\alpha (\epsilon )>0$ which depends on the behavior of *f* so that for any $t\ge 1$, ${\mathbb {P}}(m(t)> \epsilon +\min _{{\textbf{x}}\in {\mathcal {D}}}f({\textbf{x}}))<(1-\alpha (\epsilon ))^{t-1}.$

### Proof

Let ${\textbf{x}}_*$ denote a minimizer of *f*, which is an interior point of ${\mathcal {D}}$. Without loss of generality, we assume $f({\textbf{x}}_*)=0$. By (C3), $m(t):=\min _{1 \le i \le n} f({\textbf{x}}_i(t))$ is a non-increasing sequence, thus it suffices to show that $m(t) {\mathop {\rightarrow }\limits ^{P}} 0$ as $t \rightarrow \infty$. Given a fixed $\epsilon >0$, by continuity of *f*, there exists $\delta >0$ such that $0 \le f({\textbf{x}}) \le \epsilon$ for ${\textbf{x}}\in B({\textbf{x}}_*, \delta ):=\{{\textbf{x}}:\Vert {\textbf{x}}-{\textbf{x}}_*\Vert \le \delta \}$. Since ${\textbf{x}}_*$ is an interior point, $B({\textbf{x}}_*, \delta ) \subset {\mathcal {D}}$ for a sufficiently small $\delta$. By (C2), at each iteration, there is an agent ${\textbf{x}}_i$ such that either $f({\textbf{x}}_i(t))\le \epsilon$ or ${\mathbb {P}}_t\left( {\textbf{x}}_{i(t+1)}(t+1)\in B({\textbf{x}}_*, \delta ) \right) \ge \alpha | B({\textbf{x}}_*, \delta ) | =: \alpha (\epsilon ).$ Note that $\alpha (\epsilon )=\alpha |B({\textbf{x}}_*, \delta )|\le 1$ when (C2) holds. If $f({\textbf{x}}_i(t))\le \epsilon$, then $m(t)\le \epsilon$. If ${\textbf{x}}_{i(t+1)}(t+1)\in B({\textbf{x}}^*, \delta )$, then $m(t+1)\le f({\textbf{x}}_{i(t+1)}(t+1))\le \epsilon$. By the tower property of conditional expectation,

4 $$\begin{aligned} {\mathbb {P}}(m(t)> \epsilon )&\le {\mathbb {P}}({\textbf{x}}_{i(s+1)}(s+1) \notin B({\textbf{x}}^*, \delta ) , \text { for } s=1,\ldots , t-1) \end{aligned}$$

5 $$\begin{aligned}&={\mathbb {E}}\prod _{s=1}^{t-1}{\mathbb {P}}_s({\textbf{x}}_{i(s+1)}(s+1)\notin B({\textbf{x}}^*, \delta ) ) \le {\mathbb {E}}\prod _{s=1}^{t-1}(1-\alpha (\epsilon ))=(1-\alpha (\epsilon ))^{t-1}. \end{aligned}$$

Therefore, $$m(t) {\mathop {\rightarrow }\limits ^{P}} 0$$ as $$t\rightarrow \infty$$. $$\square$$

Theorem 1 shows that any swarm-based algorithm satisfying (C1)–(C3) is guaranteed to converge to a neighborhood of global optimum after finitely many iterations. Many swarm-based algorithms satisfy (C3), but not all swarm-based algorithms stay within the search space ${\mathcal {D}}$ in their iterations thus violating condition (C1) or they are exploratory in the sense of condition (C2). For example, if an agent of the PSO swarm is initialized near the boundary of ${\mathcal {D}}$ and the momentum points beyond ${\mathcal {D}}$, this agent will likely leave ${\mathcal {D}}$ in the next iteration. Furthermore, if the swarm agents arrive at the same location, say a suboptimal local optimum, with zero momentum, the algorithm will stay at this location in all subsequent iterations^9,10^. However, “Perturbation-projection strategy” section below shows that, with slight modifications of an algorithm, we can ensure the modified algorithm satisfies (C1)–(C2).

Our result actually provides a finite iteration analysis. In particular, given an error tolerance, $\epsilon >0$, and probability tolerance, $\delta >0$, Theorem 1 says if $t>t_0 = \left\lceil \frac{\log \delta }{\log (1-\alpha (\epsilon ))}\right\rceil$ (which is a finite number), the algorithm at iteration $t_0$ can find the global optimum with $\epsilon$ accuracy with probability at least $1-\delta$. Such form of finite iteration analyses can also be found for other stochastic algorithms in machine learning, for example the stochastic gradient desecent^33^.

### Perturbation-projection strategy

A first step is to ensure all agents stay in ${\mathcal {D}}$. For this purpose, we introduce the projection onto ${\mathcal {D}}$ ensuring the agents stay inside the search space, ${\mathcal {D}}$, a compact subset in ${\mathbb {R}}^d$:

$$\begin{aligned} \chi _{\mathcal {D}}({\textbf{x}})=\arg \min _{{\textbf{y}}\in {\mathcal {D}}} \Vert {\textbf{y}}-{\textbf{x}}\Vert _2, \end{aligned}$$

where $\Vert {\textbf{y}}-{\textbf{x}}\Vert _2$ denotes the Euclidean distance between ${\textbf{x}}$ and ${\textbf{y}}$. In other words, $\chi _{\mathcal {D}}({\textbf{x}})$ is a point in ${\mathcal {D}}$ that is closest to ${\textbf{x}}$. When ties occur, they can be broken arbitrarily. By definition, $\chi _{\mathcal {D}}({\textbf{x}})={\textbf{x}}$ if ${\textbf{x}}\in {\mathcal {D}}$. In general, adding the projection to an algorithm’s iterations reduces the agents’ distances to the optimal solution^33^. In particular, if ${\textbf{x}}_*\in {\mathcal {D}}$ and ${\mathcal {D}}$ is convex, it can be shown that $\Vert \chi _{\mathcal {D}}({\textbf{x}})-{\textbf{x}}_*\Vert \le \Vert {\textbf{x}}-{\textbf{x}}_*\Vert _2.$ In many optimization problems, the search space, ${\mathcal {D}}$, is usually a *d*-dimensional hyper-rectangle and it follows that ${\mathcal {D}}$ is convex.

A second step is to enhance the algorithm’s exploration ability by injecting noise into its dynamics. In stochastic optimization algorithms, such as the perturbed gradient descent and the Langevin algorithm, Gaussian noise is added to the classical gradient descent algorithm. This injection of noise effectively helps the algorithm to get out of saddle points or local minimums efficiently^34,35^. We adopt here the same strategy due to its simplicity.

By combining these two steps, the modified algorithm can be formulated as

6 $$\begin{aligned} {\textbf{x}}_{i}(t+1)=\chi _{\mathcal {D}}(\chi _{\mathcal {D}}({\textbf{x}}'_i(t+1))+{\textbf{w}}_i(t)), \end{aligned}$$

where ${\textbf{x}}'_i(t+1)$ is the position of the *i*th agent after applying the algorithm’s update rule at $t+1$, and ${\textbf{w}}_i(t)$ is an independent draw from a density $p_t$ such that there is a constant $\alpha >0$

7 $$\begin{aligned} p_t({\textbf{y}}-{\textbf{x}})>\alpha ,\quad \text{ for } {\textbf{x}},{\textbf{y}}\in {\mathcal {D}}. \end{aligned}$$

The noise density $p_t$ in general can be adaptive, depending on the evolution of the algorithm up to time *t*. Here we only need it to be strictly positive and bounded away from zero. We call this method of modification in (6) a perturbation-projection (PP) modification or strategy. The proposition below shows that with PP strategy, the modified algorithm satisfies (C1) and (C2).

#### Proposition 2

Suppose *A* is a swarm-based algorithm and at each iteration, every agent is projected onto ${\mathcal {D}}$. If the perturbation-projection modification (6) is applied to at least one of the agents, then *(C1)* and (C2) hold.

#### Proof

Since $\chi _{{\mathcal {D}}}$ is applied to all agents, (C1) clearly holds. At the $(t+1)$th iteration, denote the agent to which the modification is applied by $i=i(t+1)$. Let ${\textbf{y}}=\chi _{\mathcal {D}}({\textbf{x}}'_{i}(t+1))$.

Then, for any *d*-dimensional ball *B* in ${\mathcal {D}}$,

8 $$\begin{aligned} \max _{1 \le k \le n}&{\mathbb {P}}_t&( {\textbf{x}}_k(t+1) \in B )\ge {\mathbb {P}}_t({\textbf{x}}_{i(t+1)}(t+1)\in B)\end{aligned}$$

9 $$\begin{aligned}\ge & {} {\mathbb {P}}_t({\textbf{y}}+{\textbf{w}}_i(t)\in B) =\int _B {\mathbb {P}}_t({\textbf{y}}+ {\textbf{w}}_i(t)\in d{\textbf{x}}) =\int _B {\mathbb {P}}_t({\textbf{w}}_i(t) \in d({\textbf{x}}-{\textbf{y}})) =\int _B p_t({\textbf{y}}-{\textbf{x}})d{\textbf{x}}\ge \alpha |B|, \end{aligned}$$

showing (C2) holds. $$\square$$

## Applications

Before we proceed to illustrate how to implement our PP strategy in Theorem 1 to three nature-inspired swarm-based optimization algorithms, namely, particle swarm optimization (PSO), bat algorithm (BAT) and ant colony optimization for continuous domains (ACO), we incoproate an observation from nature. It is common to observe that agents in a swarm in nature are often divided to specialize in different tasks and they collaborate to improve collective effectiveness. Inspired by this, we set some agents in the swarm to specialize in exploration while others in exploitation. In the context of PP strategy, we only perturb the exploration agents so they help exploring the search space while we do not perturb exploitation agents so as not to hamper their convergence capability. We call this heterogeneous perturbation-projection, abbreviated to HPP, strategy. Proposition 2, which only requires the perturbation strategy applied to at least one agent, continues to hold for algorithms with HPP strategy. Consequnetly, Theorem 1 guarantees HPP modified algorithms converge a.s to a global optimal solution. We denote the modified version of an algorithm *A* using HPP strategy by *hmA*. We also summarize its formulation as Pseudo code 1. For the present work, the proportions of exploration agents and exploitation agents are taken to be 1/2. One can treat the proportion of exploration agents as an additional parameter in an algorithm to be tuned, and this will be left for future work.

Our HPP strategy can be considered as a special case of cooperative or collaborative learning, which suggests that mixing agents of different responsibilities can lead to improved overall performance. Existing works on cooperative swarm-based algorithms can be found in^36–38^. In comparison, our HPP strategy focuses more on the cooperation between exploration agents and exploitation agents. Recently,^39^ also applies this idea to local optimization algorithms such as gradient descent and Langevin algorithm.
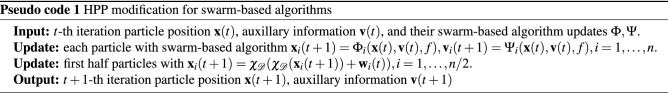


### PSO and hmPSO

In^40^, Kennedy and Eberhart proposed the particle swarm optimization (PSO) algorithm. The algorithm has stimulated many refinements and inspired many variants, and these algorithms find wide applications in many fields. A recent search of “particle swarm optimization” in the Web of Science generated more than to 32,000 articles and almost 600 review articles. A small sampling of early examples of how PSO has been studied and modified over the years are^36,41–45,45–48^. PSO probably has the most modified versions among nature-inspired metaheuristic algorithms and they continue to this day.

PSO algorithm models after the movement of a flock of birds looking for food collectively. To align with our terminology in this article, we think of birds in PSO as agents, and food as a global minimizer of an objective function. At the next iteration, $t+1$, each agent veers towards the best location it has found (cognitive/memory component) and the best location known to the flock up to iteration *t* (social component). To describe the memory effect, we allocate a personal “memory” agent ${\textbf{x}}^*_i(t)$ to record the best location agent *i* has found up to iteration *t*, and a global “memory” agent ${\textbf{x}}^*(t)$ to record the best location found by all agents collectively so far. Recall the basic PSO algorithm runs the following steps iteratively:

1. Generate two independent random vector ${\textbf{U}}_1$ and ${\textbf{U}}_2$ from the uniform distribution on $[0,1]^d$ and let
  10 $$\begin{aligned} {\textbf{v}}_i(t+1)=w{\textbf{v}}_i(t)+c_1{\textbf{U}}_{1}\circ ({\textbf{x}}_i^*(t)-{\textbf{x}}_i(t)) + c_2 {\textbf{U}}_{2}\circ ({\textbf{x}}^*(t)-{\textbf{x}}_i(t)), \end{aligned}$$
  where $w,c_1, c_2$ are given constants, and $\circ$ denotes the Hadamard product.
2. Update $${\textbf{x}}_i(t+1)={\textbf{x}}_i(t)+{\textbf{v}}_i(t+1)$$.
3. Update personal best
  11 $$\begin{aligned} {\textbf{x}}^*_i(t+1)={\left\{ \begin{array}{ll} {\textbf{x}}_i(t+1), &{}\text{ if } f({\textbf{x}}^*_i(t))>f({\textbf{x}}_{i}(t+1));\\ {\textbf{x}}^*_i(t), &{}\text{ otherwise }. \end{array}\right. } \end{aligned}$$
4. Repeat steps 1–3 for all agents, then update the global best agent
  12 $$\begin{aligned} {\textbf{x}}^*(t+1)={\left\{ \begin{array}{ll} {\textbf{x}}_j^*(t+1), &{} \text{ if } \text{ condition } \text{ H } \text{ holds }, \\ {\textbf{x}}^*(t), &{}\text{ otherwise }, \end{array}\right. } \end{aligned}$$
  where $j=\text {argmin}_{1\le i\le n} f({\textbf{x}}^*_i(t+1))$, $m(t)= \min _{1\le i \le n} f({\textbf{x}}^*_i(t))$ and condition H: $m(t+1) < m(t)$.

Our modified PSO, denoted by hPSO, is to apply the HPP strategy in step 2 only to the exploration agents (labelled as $1, \ldots , n/2$):

2’) Update, for $$1 \le i \le n/2$$,

13 $$\begin{aligned} {\textbf{x}}_i(t+1)=\chi _{{\mathcal {D}}}\left( \chi _{{\mathcal {D}}}\left( {\textbf{x}}_i(t)+{\textbf{v}}_i(t+1)\right) +{\textbf{w}}_i(t)\right) , \end{aligned}$$

where ${\textbf{w}}_i$’s are independent multivariate normal distributed with mean vector ${\textbf{0}}$ and covariance matrix $\sigma {\textbf{I}}$.

Since its introduction in 1995, there have been numerous attempts to analyze the basic PSO convergence behavior. In our opinion, Yuan and Yin provided in^49^ the first rigorous proof of the weak convergence of PSO to a global optimum, without overly restrictive assumptions. Moreover, using stochastic approximation technique, they provide the rate of convergence. In another direction,^50^ introduced two smoothed versions of PSO and prove that they converge almost surely to a cost function’s global optimum.

The next proposition shows that if we modify the basic PSO algorithm by replacing the update step 2 by the new noise enhanced update step 2’, the modified mPSO algorithm converges to a global minimum of the cost function almost surely

#### Proposition 3

Let *f* be a continuous function defined on a compact subset ${\mathcal {D}}$ of ${\mathbb {R}}^d$ and one of its minimizers is in the interior of ${\mathcal {D}}$. Then the algorithm hmPSO converges to $\min _{{\textbf{x}}\in {\mathcal {D}}} f({\textbf{x}})$ almost surely.

#### Proof

By Theorem 1, it suffices to verify that hmPSO satisfies (C1)–(C3). Note that ${\textbf{x}}_i(t+1) \in {\mathcal {D}}$ by projection. As ${\textbf{x}}_i^*(t+1)$ takes either value ${\textbf{x}}_i^*(t)$ or ${\textbf{x}}_i(t+1)$, therefore ${\textbf{x}}_i^*(t+1) \in {\mathcal {D}}$ if ${\textbf{x}}_i^*(t)\in {\mathcal {D}}$. Similar argument applies to ${\textbf{x}}^*(t+1)$, so (C1) holds by induction. Since we add noise to the exploration agents ${\textbf{x}}_i$ ($1 \le i \le n/2$). Proposition 2 implies (C2) holds. Consider the global memory agent, by step 4, $f({\textbf{x}}^*(t+1)) \le f({\textbf{x}}^*(t))$, so (C3) is verified. This completes the proof. $\square$

### BAT and hmBAT

The success of bats, dolphins, shrews and other animals in applying echolocation technique to hunt and navigate inspired^51^ to propose the Bat Algorithm (BAT) in 2010. BAT meets with rising popularity in applications. According to a recent search of the Web of Science, over 1,500 articles, nearly 800 proceedings papers and 48 review papers have been written on this algorithm and its variants in just ten years. We refer interested readers in BAT and its wide ranging applications to the review articles in^52,53^ and the references therein. Recent applications of the BAT algorithm include^54,55^. As with other metaheuristic algorithms, BAT has many modified or hybridized versions for improved performance in various ways; some examples of the modified versions can be found in^56–60^ and some examples of BAT algorithm hybridized with another metaheuristic algorithm are^61–63^.

Let ${\textbf{x}}_i(t)$, $1 \le i \le n$, denote the position of the *i*-th bat at *t*-th iteration, and ${\textbf{x}}^*(t)$ tracking the best position found by the cauldron of *n* bats. Recall BAT runs the following iterations iteratively:

1. Generate *n* independent $U_i$ from the uniform distribution on $[f_{\min },f_{\max }]$, ${\textbf{v}}_i(t+1)= {\textbf{v}}_i(t)+U_{i} ({\textbf{x}}_i(t)-{\textbf{x}}^*(t)).$
2. Let $r_0$ (pulse rate) denote a user-specified threshold. Generate *n* independent random numbers $r_i$ from the uniform distribution on [0, 1], $1\le i \le n$. Update according to (a) $r_i < r_0$ or (b) $r_i \ge r_0$:
  - If $$r_i < r_0$$, $${\textbf{x}}_i(t+1)={\textbf{x}}_i(t)+{\textbf{v}}_i(t+1)$$.
  - If $r_i \ge r_0$, ${\textbf{x}}_i(t+1)={\textbf{x}}^*(t)+\epsilon _i(t)$, where the components of $\epsilon _i(t)$ are independent mean 0 normal distributions with standard deviation 0.001.
3. Generate *n* independent random numbers $r_i$ from the uniform distribution on [0, 1]. If $r_i$ is less than a threshold $r_A$ (loudness), or $f(\chi _{{\mathcal {D}}}({\textbf{x}}_i(t)))<f({\textbf{x}}_i(t+1))$ update ${\textbf{x}}_i(t+1)=\chi _{{\mathcal {D}}}({\textbf{x}}_i(t))$.
4. Update global best ${\textbf{x}}^*(t+1)={\textbf{x}}_{i^*}(t+1),$ where $i^*=\arg \min _i f({\textbf{x}}_i(t+1)).$

In the procedure above, $f_{\min }, f_{\max }$ are given constants which describe the minimal and maximal frequencies. The threshold probabilities $r_0$ and $r_A$ describe the pulse and emission rates. The random noise $\epsilon _i(t)$ describes the loudness of each bat.^51^ also suggests using time varying $\epsilon _i(t)$ and $r_0$ for improved performance. Here we fix them as in the standard values suggested by the MATLAB package provided by the same author.

Our modified BAT, denoted by hmBAT, is to apply the HPP strategy by an additional step after step 3:

3’) Update, for $$1 \le i \le n/2$$,

14 $$\begin{aligned} {\textbf{x}}_i(t+1)=\chi _{{\mathcal {D}}}\left( \chi _{{\mathcal {D}}}\left( {\textbf{x}}_i(t)+{\textbf{v}}_i(t+1)\right) +{\textbf{w}}_i(t+1)\right) . \end{aligned}$$

#### Proposition 4

Let *f* be a continuous function defined on ${\mathcal {D}}$, a compact subset of ${\mathbb {R}}^d$. Suppose further that a minimizer of *f* lies in the interior of ${\mathcal {D}}$, then the algorithm hmBAT converges to $\min _{{\textbf{x}}\in {\mathcal {D}}} f({\textbf{x}})$ almost surely.

#### Proof

By Theorem 1, it suffices to verify hmBAT satisfies (C1)–(C3). Note that ${\textbf{x}}_i(t+1) \in {\mathcal {D}}$ by projection. As ${\textbf{x}}^*(t+1)$ takes either value ${\textbf{x}}^*(t)$ or ${\textbf{x}}_i(t+1)$ for some *i*, therefore it is in ${\mathcal {D}}$ if ${\textbf{x}}^*(t)\in {\mathcal {D}}$. So (C1) holds by induction.

To verify (C2), we denote the value of ${\textbf{x}}_i(t+1)$ after step 3’) as ${\textbf{y}}_i(t)$, which is given by equation (14). Following the proof of Proposition 2, we can show that for any d-ball $B\subset {\mathcal {D}}$, ${\mathbb {P}}({\textbf{y}}_i(t)\in B)\ge \alpha |B|.$ If all ${\textbf{y}}\in B$ satisfies $f({\textbf{y}})<f({\textbf{x}}_i(t))$, we have

15 $$\begin{aligned} {\mathbb {P}}({\textbf{x}}_i(t+1)\in B)\ge (1-r_A){\mathbb {P}}({\textbf{y}}_i(t)\in B)\ge \alpha (1-r_A) |B|. \end{aligned}$$

So (C2) holds.

By step 4, $f({\textbf{x}}^*(t+1)) \le f({\textbf{x}}^*(t))$, so (C3) is verified. This completes the proof of Proposition 4. $\square$

### ACO and hmACO

Ant colony Optimization (ACO) was initially proposed to solve discrete optimization such as the traveling salesman problem^64,65^. Later, it is adapted to solve continuous optimization problems in^66^. We focused our discussion of ACO on this adaptation, since our discussion so far was mainly about continuous optimization.

We shall call the particles in ACO ants. At each iteration, ACO maintains an archive of *n* best ants ${\textbf{x}}_1(t),\ldots , {\textbf{x}}_n(t)$. Each ant is a *d*-dimensional vector with components ${\textbf{x}}_i(t)=(x_{i,1}(t),\ldots , x_{i,d}(t))$. Without loss of generality, we assume the ants are ranked so $f({\textbf{x}}_1(t))\le f({\textbf{x}}_2(t))\le \cdots \le f({\textbf{x}}_n(t))$. In the beginning of a new iteration, ACO generates *m* new ants by sampling Gaussian mixture distribution at each dimension. The Gaussian mixtures consist of Gaussian distributions centered at each archived ant with weights according to the ant’s performance. After the new ants are generated, they are inserted to the archive, and the archive will then remove *m* ants with worst performances. Specifically, ACO repeats steps 1 to 4 until termination criterion is met.

1. For each dimension *j*, construct a 1-dimensional Gaussian mixture $p_j$ density centered at $\{x_{1,j}(t),\ldots , x_{n,j}(t)\}$
  16 $$\begin{aligned} p_j(x)=\sum _{i=1}^n \frac{w_i}{\sigma _{i,j} \sqrt{2\pi }} \exp \left( -\frac{(x-x_{i,j}(t))^2}{2\sigma ^2_{i,j}}\right) . \end{aligned}$$
  The weight $w_i$ is a decreasing sequence so better performing ants are favored.^66^ suggests using
  17 $$\begin{aligned} w_i=\frac{\exp (-(i-1)^2/(2q^2 n^2)))}{\sum _{i=1}^n \exp (-(i-1)^2/(2q^2 n^2)))}, \end{aligned}$$
  with certain tuning parameter *q*. Smaller *q* will give more preference to the best performing ant ${\textbf{x}}_1$. The standard deviation is suggested to be chosen as $\sigma _{i,j}=\sigma \sum _{k=1}^n |x_{i,j}(t)-x_{k,j}(t)|/(n-1),$ with $\sigma$ being a tuning parameter.
2. Generate *m* new ants ${\textbf{y}}_{1}(t),\ldots , {\textbf{y}}_{m}(t)$ independently. Each follows the product distribution ${\textbf{y}}_i(t)\sim \prod _{j=1}^d p_j(x), i=1,\ldots ,m.$
3. Combine them with existing archived ants and reorder them. That is, we let
  18 $$\begin{aligned} \{{\textbf{x}}'_1,\ldots ,{\textbf{x}}'_{n+m}\}=\{{\textbf{x}}_1(t),\ldots , {\textbf{x}}_n(t), {\textbf{y}}_1(t),\ldots , {\textbf{y}}_m(t)\} \end{aligned}$$
  where $$f({\textbf{x}}'_1)\le f({\textbf{x}}'_2)\le \cdots \le f({\textbf{x}}'_{n+m}).$$
4. Update archive with the best ants ${\textbf{x}}_i(t+1)={\textbf{x}}'_i$ for $i=1,\ldots ,n$.

Our modified ACO, denoted by hmACO, is to apply the HPP strategy in step 2):

2’) Generate *m* new ants ${\textbf{y}}_{1},\ldots , {\textbf{y}}_{m}$ independently by first sampling from the product distribution ${\textbf{y}}'_i(t)\sim \prod _{j=1}^d p_j(x),i=1,\ldots ,m.$ Then, let

19 $$\begin{aligned} {\textbf{y}}_{i}(t)=\chi _{{\mathcal {D}}}\left( \chi _{{\mathcal {D}}}\left( {\textbf{y}}'_i(t))+{\textbf{w}}_{i}(t)\right) \right) \end{aligned}$$

and assume that the first batch of ants ${\textbf{x}}_i(0)$ are in the feasible set ${\mathcal {D}}$. Otherwise, replace them with $\chi _{{\mathcal {D}}}({\textbf{x}}_i(0))$.

We show that hmACO converges almost surely as long as $m <n/2$. This setting is relevant in practice, since the computational cost comes mainly from the new ants generation and evaluations. Intuitively, having many new ants without updating the archive will make the algorithm less efficient. For example, Socha and Dorigo^66^ suggests using $m=2$ and $n=50$.

#### Proposition 5

Let *f* be a continuous function over ${\mathcal {D}}$, a compact subset of ${\mathbb {R}}^d$. Suppose further that a minimizer of *f* lies in the interior of ${\mathcal {D}}$, then the algorithm hmACOconverges to $\min _{{\textbf{x}}\in {\mathcal {D}}} f({\textbf{x}})$ almost surely if $m<n/2$.

#### Proof

By Theorem 1, it suffices to prove that mACO satisfies (C1)–(C3). Note that ${\textbf{x}}_i(t+1)$ is either ${\textbf{y}}_j(t)$ or ${\textbf{x}}_j(t)$ for some *j*, the former is in ${\mathcal {D}}$ by projection, so (C1) holds by induction. Since $m < n$ and only *m* ants will be removed from $\{{\textbf{y}}_1(t),\ldots , {\textbf{y}}_m(t), {\textbf{x}}_1(t),\ldots , {\textbf{x}}_n(t)\}$, ${\textbf{x}}_1(t)$ remains in the archive at iteration $t+1$. This implies (C3) holds.

For any *d*-dimensional ball *B* in ${\mathcal {D}}$, let ${\mathbb {P}}_t'$ be the conditional probability given ${\textbf{y}}'_1(t),\ldots ,{\textbf{y}}'_m(t)$, then for any *j*

20 $$\begin{aligned} {\mathbb {P}}'_t( {\textbf{y}}_j(t) \in B ) \ge {\mathbb {P}}'_t(\chi _{\mathcal {D}}({\textbf{y}}'_{j}(t))+{\textbf{w}}_j(t)\in B) =\int _B p_t(\chi _{\mathcal {D}}({\textbf{y}}'_{j}(t))-{\textbf{x}})d{\textbf{x}}\ge \alpha |B|. \end{aligned}$$

Note that if $f({\textbf{y}})\le f({\textbf{x}}_1(t))$ for all ${\textbf{y}}\in B$ and ${\textbf{y}}_j(t)\in B$, we have $f({\textbf{y}}_j(t))\le f({\textbf{x}}_1(t))$ and ${\textbf{y}}_j(t)$ will be kept in the archive at iteration $t+1$. Consequently, ${\mathbb {P}}'_t( {\textbf{x}}_j(t+1) \in B \text { for some } j )\ge \alpha |B|$ and (C2) holds. $\square$

## Numerical experiments

We conduct two sets of numerical experiments, Experiment 1 and Experiment 2 to compare the perormance of *A* and *hmA* for $A=$ PSO, BAT or ACO. Details are given in “Experiment 1” and Experiment 2” sections. In “Tuning parameter analysis for HPP strategy” section, we report the analysis of tuning parameters used in HPP strategy. The main objective of our experiments is to compare the performance of *A* and *hmA* when *A* is PSO, BAT or ACO; it is not our intention to compare PSO, BAT and ACO.

The swarm size used is 32 in all the comparison studies below. At each run, we start the algorithms *A* and *hmA* with the same initialization of the particles in the swarm.

### Experiment 1

In this experiment, we conduct extensive numerical experiments to compare the performance of *A* and *hmA*, where *A* = PSO, BAT or ACO, for a computing budget on a large class of very different types of cost functions. We use two measures to evaluate how likely and by how much the modified algorithm outperforms the original algorithm.

#### Test functions and details of Experiment 1

A total of 28 test functions are used for this experiment. The functions are compiled by Wahab et al.^67^, which are of varying dimensions and their exact expressions can be found in Supplementary Table 1 in Supplementary Material: A. The list of functions includes commonly used test functions such as Ackley, Griewank, Powell, Rastrigin, Sphere with different properties. Some are unimodal, multi-modal, separable or inseparable. The majority of the functions are defined for arbitrary dimension *d*. For such functions, we investigate the effect of dimension on the optimization algorithms for selected dimensions, i.e., $d =5, 10, 20$ and 40. The other test functions have dimensions $d=2$, except for one with $d=4$, which we exlcude. In total, we have a set ${\mathcal {C}}$ of 70 test functions and we summarize the numerical results according to their dimensions, i.e., $d=2, 5, 10, 20$ and 40.

For each cost function $f \in {\mathcal {C}}$, we conduct 100 runs of the algorithms *A* and *hmA*, each for 10,000 iterations, where *A* = PSO, BAT, or ACO. To gauge the progress of each run of an algorithm, we output the algorithm’s best functional values found during the search at the *t*th iteration, where $t=50, 100, 200, 400, 1000, 3000$ and 10, 000.

For all experiments, we use $n=32$ agents/particles and for *hmA* ($A =$ PSO, BAT or ACO). We employ commonly used parameter settings for PSO, BAT and ACO, namely,

- PSO: $w=0.729$ (inertia weight), $c_1=c_2=1.5$ (acceleration constants);
- BAT: $f_{\min }=0$ (minimum frequency), $f_{\max }=100$ (maximum frequency), $r_0=0.5$ (pulse rate), $r_A=0.5$ (loudness);
- ACO: $$q=10^{-4},\sigma =0.85, n=32, m=2$$.

For *hmA*, we use the same parameters as in *A* with the stochastic perturbations being independent normal variates, each with mean 0 and standard deviation 0.005.

#### Comparison and results

This subsection compares the performance of an algorithm *A* versus one of its modification *B* using several test functions from the set ${\mathcal {C}}$, functions in the set are commonly used to compare performance of an algorithm in the engineering literature. We are particularly interested to ascertain how their performance depends on the dimension of the problem. Here, ${\mathcal {F}}$ is the subset of ${\mathcal {C}}$ that contains functions of the same dimension $d=2,5,10,20$ or 40. Our two key questions are:(a) How likely does *B* outperform *A* and (b) On average, how much does *B* outform *A* at termination, in terms of the criterion value and $B=hmA$.

(a) *How likely will* *B* *outperform* *A?*

Let $A_f(t)$ and $B_f(t)$ denote the (theoretical) best functional values from algorithms *A* and *B*, respectively, for the test function *f*, up to the *t*th iteration. Similarly, let $A_{r,f}(t)$ and $B_{r,f}(t)$ denote the best functional values from the algorithms *A* and *B*, respectively, up to the *t*th iteration at the *r*th run. The probability that algorithm *B* outperforms algorithm *A* for *f* at the *t*th iteration is ${\mathbb {P}}(B_f(t) < A_f(t))$, and it can be estimated by $\sum _{r=1}^{100} I(B_{r,f}(t) < A_{r,f}(t))/100$. The average of these estimates over ${\mathcal {F}}$, denoted by $P_{B \succ A}(t)$, can be thought of as an average winning proportion of *B* over *A* (or simply, winning proportion). Specifically,

21 $$\begin{aligned} P_{B \succ A}(t):=\frac{1}{100 |{\mathcal {F}}|} \sum _{f \in {\mathcal {F}}} \sum _{r=1}^{100} I( B_{r,f}(t) < A_{r,f}(t)). \end{aligned}$$

Similarly, interchanging *A* and *B* above, we define the winning proportion of *A* over *B*, $P_{A \succ B}(t)$. Since $P_{A \succ B}(t) + P_{B \succ A}(t) =1$, it suffices to track $P_{B \succ A}(t)$. Loosely speaking, if $P_{B \succ A}(t) >1/2$, *B* is more likely to outperform *A* for $f \in {\mathcal {F}}$. Indeed, the larger the value of $P_{B \succ A}(t)$, the more likely it is that *B* outperforms *A*.

Supplementary Figure 1 in the Supplementary Material displays the comparison results and we observe the following. When *t* is fixed, the winning proportion of HPP modified algorithm outperforming its counterpart increases as the dimension of the test function increases. Both mBAT and hmBAT are comparable in their performance against BAT, and the winning proportion increases steadily from about 0.5 to 0.7 for dimension at least 5. Interestingly, the HPP strategy has more significant enhancement effect on PSO and ACO.

(b) *How much does* *B* *(or* *A) outperform* *A* *(or* *B)?*

For simplicity, we suppress the dependence of a notation on other parameters/notations when the context is clear. To this end, let $m_*$ and $m^*$ denote respectively, the minimum (i.e., the best) and maximum (i.e., the worst) of the outputs from 100 runs of *A* and 100 runs of *B* for *f* at the *t*th iteration. We view $m_*$ as a proxy of the best possible output by *A* and *B*; and let $R = m^*-m_*$ denote the range of the 200 outputs of *A* and *B*. Define the relative error of *A* with respect to *A* and *B*, $RE_{A, A\cdot B, f}(t)$, to be the average of $(A_{r,f}(t) - m_*)/R$ over 100 runs; and the relative error of *B* relative to *A* and *B*, $RE_{B, A \cdot B, f}(t)$ is similarly defined. We consider the relative instead of absolute error in order to cancel out the effect due to a scale change of *f* (i.e., if we consider *cf* instead of *f*). For brevity, we simply call this quantity the relative error. Note that the relative errors lie between 0 and 1. Small relative error of *A* (i.e., $RE_{A, A\cdot B, f}(t)$) indicates the results from algorithm *A*, as a whole, are closer to the best possible value $m_*$. Further, if the relative error of *A* is less than that of *B*, then generally results from algorithm *A* are closer to $m_*$ than those from *B*’s. A similar interpretation can be extended over a class of functions $f \in {\mathcal {C}}$ if we define $RE(A, A\cdot B)(t):=\frac{1}{|{\mathcal {F}}|} \sum _{f \in {\mathcal {F}}} RE_{A, A \cdot B, f}(t)$ and call this the overshoot of *A* relative to *A* and *B*.

Supplementary Figure 2 in the Supplementary Material displays various performances of the PSO, BAT and ACO algorithms listed across columns relative to their modified algorithms in their first 10,000 iterations. Each row displays summarized results from test functions grouped by one of their dimensions with $d = 2, 5, 10, 20$ and 40 resulting in 5 subfigures per column. Each subfigure displays the relative error of (i) A relative to A and hmA combined (red curve), and (ii) hmA relative to A and hmA combined (blue curve). If the blue (respectively, red) curve is lower, it indicates the hmA (respectively, A) is more superior than A. The subfigures are informative. For example, from subfigures in the rows for $d=10$ and $d=40$, we observe that: (i) hmBAT outperforms BAT by a significant margin for all dimensions *d*; (ii) When $d=40$, relative errors of hmPSO are nearly 0 from 1000 iterations onwards. This implies that hmPSO significantly outperforms the basic PSO from 1000 iterations; (iii) At $d=10$, hmA outperforms A for A = PSO, BAT and ACO from 400 iterations.

Combining the observations in (a) and (b), Supplementary Figures 1 and 2 convincingly demonstrate that the HPP strategy significantly improves BAT, ACO and PSO, particularly when the dimension of the test function is large. Improvement is measured in two complementary aspects, “how likely” and “how much” one algorithm outperforms the other.

### Experiment 2

Upon the suggestion of a reviewer, we conduct numerical experiment 2 using selected benchmark functions in^68,69^. These functions are intended for the CEC2017 competition in a special session on constrained single objective real-parameter optimization. Since the focus of our paper is on unconstrained optimization, we select optimization problems in^69^ with inequality constraints for experiment 2 and transform them to unconstrained optimizations by modifying the original objective function $f({\textbf{x}})$ in the problem

$$\begin{aligned} \min \{f({\textbf{x}}): {\textbf{x}}\in [-a, a]^d\} ~ \text{ such } \text{ that } g_k({\textbf{x}}) \le 0, ~ \text{ for } 1 \le k \le K; \end{aligned}$$

to one with the objective function $f_+({\textbf{x}})$ given by

22 $$\begin{aligned} f_+({\textbf{x}}) = {\left\{ \begin{array}{ll} f({\textbf{x}}), &{} \text{ if } g_k({\textbf{x}}) \le 0, 1 \le k \le K, \\ +\infty , &{} \text{ otherwise }. \end{array}\right. } \end{aligned}$$

#### Benchmark functions and details of Experiment 2

Benchmark constrained optimization problems, C01, C02, C04, C05 and C20 in^69^ are chosen for comparing the performance of *A* and *hmA* where $A =$ PSO, BAT or $ACO$ using the device in (22), who also introduced random translation, ${\textbf{x}}-{\textbf{o}}$, followed by random rotations of the translated variables by randomly generated orthogonal matrices, in addition to the constrained optimization.

Throughout the comparisons, swarm size used is 32. At each run of *A* and *hmA*, we start them with the same initialization. Parameters used for PSO, hmPSO, BAT, hmBAT, ACOand hmACOfollow that in “Test functions and details of Experiment 1” section. Dimension, *d*, of the objective functions for comparison are chosen to be 10, 30, 50 and 100.

#### Results

Following^69^, we tabulate the algorithm’s best, median, worst, average, standard deviations function values at the 5000th and 10,000th iterations out of 100 runs of the algorithms. The results, rounded to three decimal places, are presented in Supplementary Tables 3, 4 and 5 in the Supplementary Material, respectively, for comparing PSO veruse hmPSO, BAT versus hmBAT and ACO  versus hmACO. After rounding the outputs from the algorithms in each run at either 5000th or 10,000th iteration, we compare which algorithm, *A* or *hmA*, gives better result (ties are ignored) and give the winning algorithm one vote. The rows, labeled as ‘Wins’, report the total winning votes of an algorithm. Entries that are in boldface denote the better results. Some of the entries are reported as ‘Inf’ due to one or more of the constraints are not satisfied and hence the corresponding $f_+$ takeing infinity as the function value.

We have the following general observations from the results. (i) From Supplementary Table 3, hmPSO outperforms PSO substantially, particularly so when the dimension of the benchmark function increases. Notably, the median and the mean of hmPSO best function values, even at halfway point, are substantially better, suggesting hmPSO give more consistent results across runs. The consistency of hmPSO results across runs of the algorithm can also be seen from the standard deviations, which are markedly smaller than those of PSO. (ii) Above observations also apply to hmACO as compared with ACO  from Supplementary Table 5 though not as striking as in the hmPSO case. For benchmark function C20, the two algorithms, hmACOand ACO, are close in terms of their performance. There are also runs in which both ACO  and hmACO  do not satisfy some of the constraints in the optimization of benchmark functions C01 and C02. (iii) From Supplementary Table 4, hmBAT marginally outperforms BAT.

### Tuning parameter analysis for HPP strategy

As demonstrated in “Comparison and results” section, having an additional stochastic component in a modified swarm-based algorithm can enhance the exploration ability of the algorithm. The stochastic component chosen is Gaussian with mean 0 and standard deviation $\sigma =0.005$. We expect the noise level, as indicated by the value of $\sigma$ or a heavier-tailed distribution, affects the efficiency of the modified algorithm since strong level of noise interferes the algorithm’s exploitation ability. We conduct further numerical experiments with the same set-up as above to investigate the effects of $\sigma$ in the Gaussian noise or having *t*-distributions that have heavier tails than the Gaussian on the performance of *mA* and *hmA*.

(i) *On the standard deviation of the Gaussian noise*  We tried $\sigma =0.005, 0.01, 0.02$ and 0.05. The plots of $P_{B\prec A}(t)$ (analogous to Supplementary Figure 1 and $RE(A, A\cdot B)(t)$ (analogous to Supplementary Figure 2) are very similar for *hmA* ($A=$ PSO, BAT and ACO) for all the $\sigma$s; and $\sigma =0.005$ and 0.01. Hence, we recommend using $\sigma$ in [0.005, 0.01]. Due to space consideration, these plots are given in Supplementary Figure 3 of the Supplementary Material.
(ii) *On the choice of distribution*   We examine the effect on the performance of the modified algorithms if we change the Gaussian distribution with a heavier-tailed distribution. We choose distributions $0.01 \sqrt{(m-2)/m} \ t_m$ for $m = 5, 10, 30$ and 60 where $t_m$ denotes the *t*-distribution with *m* degrees of freedom. The scaling factor $0.01 \sqrt{(m-2)/m}$ is to ensure the scaled *t*-distribution has the same standard deviation as that of the Gaussian noise in (i) above. Note also that $t_m$ converges in distribution to a standard normal and the scaling factor to 1 as *m* approached to infinity. For *m* large, we do not expect essential difference between the *t*-distribution and the Gaussian noise.

The analogous plots of $P_{B\prec A}(t)$ and $RE(A, A\cdot B)(t)$ using *t*-distributions for the choices of degrees of freedom look almost the same as the corresponding plots using Gaussian noise. These plots are not included in this article due to space constraints. Interested readers can view these plots in Supplementary Figure 4 of the Supplementary Material.

## Conclusions

Metaheuristic algorithms have found wide ranging applications in optimization problems. However, majority of these algorithms have no guarantee of converging to a global optimum, which is a desirable feature to have. In this paper, we propose an intuitive modification of an algorithm to ensure this global convergence capability. Moreover, we provide a rigorous mathematical proof of this guarantee. We first delineate in Theorem 1 sufficient conditions, namely (C1)–(C3), for a swarm-based algorithm to converge almost surely to a global optimum of an objective function. The class of objective functions for which Theorem 1 holds is very large. A heterogeneous perturbation-projection (HPP) strategy is then proposed to modify a given swarm-based algorithm to ensure the sufficient conditions (C1)–(C3) hold, and hence Theorem 1 follows, guaranteeing the modified algorithm converges almost surely to a global optimum. The proposed strategy is natural and simple to implement yet not algorithm-specific. We also demonstrated how this strategy is applied to PSO, Bat and ACO algorithms. Extensive numerical experiments were conducted and the results show that the modified algorithm either outperforms or performs on par with the original algorithm over a finite computational budget. Indeed, our method of proof leads to a form of finite iteration analyses (see the paragraph before “Perturbation-projection strategy” section). The tuning parameter analyses in “Tuning parameter analysis for HPP strategy” section suggest that applying the HPP strategy with mean 0 and standard deviation 0.005 Gaussian distribution for the additional stochastic component helps to improve swarm-based algorithms. We contend that even though we have proved the proposed algorithms theoretically converge to the global optimum, the numerics may not, in practice, always converge to the global minimum for all problems. Our theoretical results only discuss unconstrained optimization problems, which already include a wide range of practical applications. Additionally, we show in the second numerical experiment that the proposed algorithms can also be applied to inequality constrained optimization problems through a simple conversion.

We conclude this paper with an interesting observation from the results of our two experiments: When *A* outperforms *hmA*, *A* frequently only produces a marginally better result; however, when *hmA* outperforms *A*, the modified version quite often outperforms *A* by a relatively large margin. We offer a heuristic argument for such plausibility. When both *A* and *hmA* are exploring in the same neighbourhood of a local minimum, the stochastic element hinders the exploitation by a small amount and hence *A* is more likely to produce marginally better solution. However, when the occasion arises for *hmA* to make a big jump, thus leaving a local minimum’s neighbourhood, *hmA* has the potential to explore a better region and hence produces noticeably better solution than the original algorithm does. This observation reinforces the idea that adding noise appropriately to metaheuristic algorithms can enhance their performance.

### Consent for participate

All authors have given consent to submit to this journal for review.

## Supplementary Information

Supplementary Information.

## Data Availability

The datasets used and/or analysed during the current study available from the corresponding author on reasonable request.

## Code availability

Will be provided whent he paper is accepted.
