# Supplementary material for: Appropriate noise addition to metaheuristic algorithms can enhance their performance
Source: Sci Rep. 2023 Mar 31;13:5291. doi: 10.1038/s41598-023-29618-5 (PMC10066303; doi:10.1038/s41598-023-29618-5)
Supplement: Supplementary file 1 — Supplementary Information. [file 41598_2023_29618_MOESM1_ESM.pdf]

Appendix: A

Label	Name	min f(x)	Uni-modal	Separable
F1	Ackley	0	No	No
$-20\exp(-0.2\sqrt{\frac{1}{n}\sum_{i=1}^{n}x_i^2})-\exp(\frac{1}{n}\sum_{i=1}^{n}\cos(2\pi x_i))+20+e$				
F2	Bohachevsky2	0	No	No
$\sum_{i=1}^{d-1}\left[x_i^2+2x_{i+1}^2-0.3\cos(3\pi x_i)\cos(4\pi x_{i+1})+0.3\right]$				
F3	Bohachevsky3	0	No	No
$\sum_{i=1}^{d-1}\left[x_{i+1}^2+2x_{i+2}^2-0.3\cos(3\pi x_i+4\pi x_{i+1})+0.3\right]$				
F4	Bukin6	0	No	No
$100\sqrt{\lvert x_2-0.01x_1^2\rvert}+0.01\lvert x_1+10\rvert$				
F5	DropWave	-1	No	No
$-(1+\cos(12\sqrt{x_1^2+x_2^2}))/(0.5(x_1^2+x_2^2)+2)$				
F6	Eggholder	-959.6407	No	No
$-(x_2+47)\sin(\sqrt{\lvert x_2+\frac{x_1}{2}+47\rvert})-x_1\sin(\sqrt{\lvert x_1-(x_2+47)\rvert}))$				
F7	GoldSteinPrice	3	No	No
$[1+(x_1+x_2+1)^2(19-14x_1+3x_1^2-14x_2+6x_1x_2+3x_2^2)]\times[30+(2x_1-3x_2)^2(18-32x_1+12x_1^2+4x_2-36x_1x_2+27x_2^2)]$				
F8	Griewank	0	No	No
$1+\sum_{i=1}^{d}\frac{x_i^2}{4000}-\prod_{i=1}^{d}\cos(\frac{x_i}{\sqrt{i}})$				
F9	McCormick	-1.9133	No	No
$\sin(x_1+x_2)+(x_1-x_2)^2-1.5x_1+2.5x_2+1$				
F10	Schaffer2	0	No	No
$0.5+\frac{\sin^2(x_1^2-x_2^2)-0.5}{(1+0.001(x_1^2+x_2^2))^2}$				
F11	Schaffer4	0.292579	No	No
$0.5+\frac{\cos^2(\sin(\lvert x_1^2-x_2^2\rvert))-0.5}{(1+0.001(x_1^2+x_2^2))^2})$				
F12	Bohachevsky1	0	No	Yes
$\sum_{i=1}^{d-1}x_i^2+2x_{i+1}^2-0.3\cos(3\pi x_i)-0.4\cos(4\pi x_{i+1})+0.7$				
F13	Booth	0	No	Yes
$(x_1+2x_2-7)^2+(2x_1+x_2-5)^2$				
F14	Branin	0.397887	No	Yes
$(x_2-\frac{5.1}{4\pi^2}x_1^2+\frac{5}{\pi}x_1^2-6)^2+10(1-\frac{1}{8\pi}\cos x_1+10)$				
F15	Michalewicz5	-4.687658	No	Yes
$-\sum_{i=1}^{5}\sin(x_i)\sin^{20}(\frac{ix_i^2}{\pi})$				
F16	Rastrigin	0	No	Yes
$10d+\sum_{i=1}^{d}[x_i^2-10\cos(2\pi x_i)]$				
F17	Shubert	-186.73	No	Yes
$\prod_{j=1}^{2}(\sum_{i=1}^{5}i\cos((i+1)x_j+i))$				
F18	Beale	0	Yes	No
$(1.5-x_1+x_1x_2)^2+(2.25-x_1+x_1x_2^2)^2+(2.625-x_1+x_1x_2^3)^2$				
F19	DixonPrice	0	Yes	No
$(x_1-1)^2+\sum_{i=2}^{d}i(2x_i^2-x_{i-1})^2)$				
F20	Easom	-1	Yes	No
$-\cos(x_1)\cos(x_2)\exp(-(x_1-\pi)^2-(x_2-\pi)^2))$				
F21	Matyas	0	Yes	No
$0.26(x_1^2+x_2^2)-0.48x_1x_2$				
F22	Powell	0	Yes	No
$\sum_{i=1}^{d/4}[(x_{4i-3}+10x_{4i-2})^2+5(x_{4i-1}-x_{4i})^2+(x_{4i-2}-2x_{4i-1})^4+10(x_{4i-3}-x_{4i})^4]$				
F23	Rosenbrock	0	Yes	No
$\sum_{i=1}^{d-1}[100(x_{i+1}-x_i^2)^2+(x_i-1)^2]$				
F24	Schwefel	-418.9829d	Yes	No
$418.9829d-\sum_{i=1}^{d}x_i\sin(\sqrt{\lvert x_i\rvert})$				
F25	Trid6	-d(d+4)(d-1)/6	Yes	No
$\sum_{i=1}^{d}(x_i-1)^2-\sum_{i=1}^{d}x_ix_{i-1}$				
F26	Zakharov	0	Yes	No
$\sum_{i=1}^{d}x_i^2+(\sum_{i=1}^{d}0.5ix_i)^2+(\sum_{i=1}^{d}0.5ix_i)^4$				
F27	Sphere	0	Yes	Yes
$\sum_{i=1}^{d}x_i^2$				
F28	Sumsquare	0	Yes	Yes
$\sum_{i=1}^{d}ix_i^2$				

Supplementary Table 1. Table of test functions with their label, name, known minimum value, uni-modality and separability. Functions F4, 5, 6, 7, 9, 10, 11, 13, 14, 17, 18, 20, 21 have dimension 2, and F15 is dimension 5. All other functions were tested at dimensions 5, 10, 20 and 40.

Appendix: B

Five benchmark functions taken from CEC2017 Competition and Special Session on Constrained Single Objective Real-Parameter Optimization for numerical experiment 2.

C01	Minimize	$f(\mathbf{x}) = \sum_{i=1}^{d} \left(\sum_{j=1}^{i} z_j\right)^2$, where $\mathbf{z} = \mathbf{x} - \mathbf{o}$, $\mathbf{x} \in [-100, 100]^d$.
	Subject to	$g(\mathbf{x}) = \sum_{i=1}^{d} \left[z_i^2 - 5000\cos(0.1\pi z_i) - 4000\right] \leq 0$,
C02	Minimize	$f(\mathbf{x}) = \sum_{i=1}^{d} \left(\sum_{j=1}^{i} z_j\right)^2$, where $\mathbf{z} = \mathbf{x} - \mathbf{o}$, $\mathbf{y} = \mathbf{M}\mathbf{z}$, $\mathbf{x} \in [-100, 100]^d$.
	Subject to	$g(\mathbf{x}) = \sum_{i=1}^{d} \left[y_i^2 - 5000\cos(0.1\pi y_i) - 4000\right] \leq 0$.
C04	Minimize	$f(\mathbf{x}) = \sum_{i=1}^{d} \left[z_i^2 - 10\cos(2\pi z_i) + 10\right]$, where $\mathbf{z} = \mathbf{x} - \mathbf{o}$, $\mathbf{x} \in [-10, 10]^d$.
	Subject to	$g_1(\mathbf{x}) = -\sum_{i=1}^{d} z_i \sin(2z_i) \leq 0$,
		$g_2(\mathbf{x}) = \sum_{i=1}^{d} z_i \sin(z_i) \leq 0$.
C05	Minimize	$f(\mathbf{x}) = \sum_{i=1}^{d-1} \left[100(z_i^2 - z_{i+1})^2 + (z_i - 1)^2\right]$,
	where	$\mathbf{z} = \mathbf{x} - \mathbf{o}$, $\mathbf{y} = \mathbf{M}_1\mathbf{z}$, $\mathbf{w} = \mathbf{M}_2\mathbf{z}$, $\mathbf{x} \in [-10, 10]^d$.
	Subject to	$g_1(\mathbf{x}) = \sum_{i=1}^{d} \left[y_i^2 - 50\cos(2\pi y_i) - 40\right] \leq 0$,
		$g_2(\mathbf{x}) = \sum_{i=1}^{d} \left[w_i^2 - 50\cos(2\pi w_i) - 40\right] \leq 0$.
C20	Minimize	$f(\mathbf{x}) = g(y_d, y_1) + \sum_{i=1}^{d-1} g(y_i, y_{i+1})$,
	where	$\mathbf{y} = \mathbf{x} - \mathbf{o}$, $\mathbf{x} \in [-100, 100]^d$ and $g(u,v) = 0.5 + \frac{\sin^2\left(\sqrt{u^2+v^2}\right) - 0.5}{\left(1 + 0.001\sqrt{u^2+v^2}\right)^2}$.
	Subject to	$g_1(\mathbf{x}) = \cos^2\left(\sum_{i=1}^{d} y_i\right) - 0.25\cos\left(\sum_{i=1}^{d} y_i\right) - 0.125 \leq 0$,
		$g_2(\mathbf{x}) = \exp\left\{\cos\left(\sum_{i=1}^{d} y_i\right)\right\} - \exp(0.25) \leq 0$.

Supplementary Table 2. Benchmark functions for numerical experiment 2. We use $\mathbf{x} = (x_1, \ldots, x_d)$ to denote a position in the search space, $[-a, a]^d$; $\mathbf{z}$ a random translation of $\mathbf{x}$ by $\mathbf{o} \sim$ Uniform $[-4a/5, 4a/5]^d$. Also, $\mathbf{y}, \mathbf{w}$ denote a random rotation of $\mathbf{x} - \mathbf{o}$, i.e., $\mathbf{y} = \mathbf{M}(\mathbf{x} - \mathbf{o})$ where $\mathbf{M}$ is orthogonal.

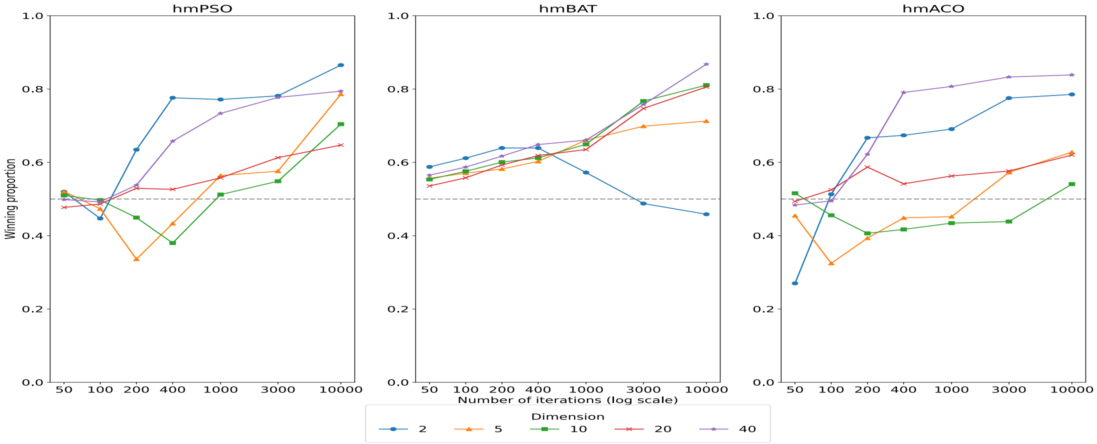

Supplementary Figure 1. Plots of average winning proportion of *mA* against *A* (top row), and that of *hmA* against *A* (bottom row) where $A =$ PSO, BAT and ACO using test functions grouped by their dimensions.

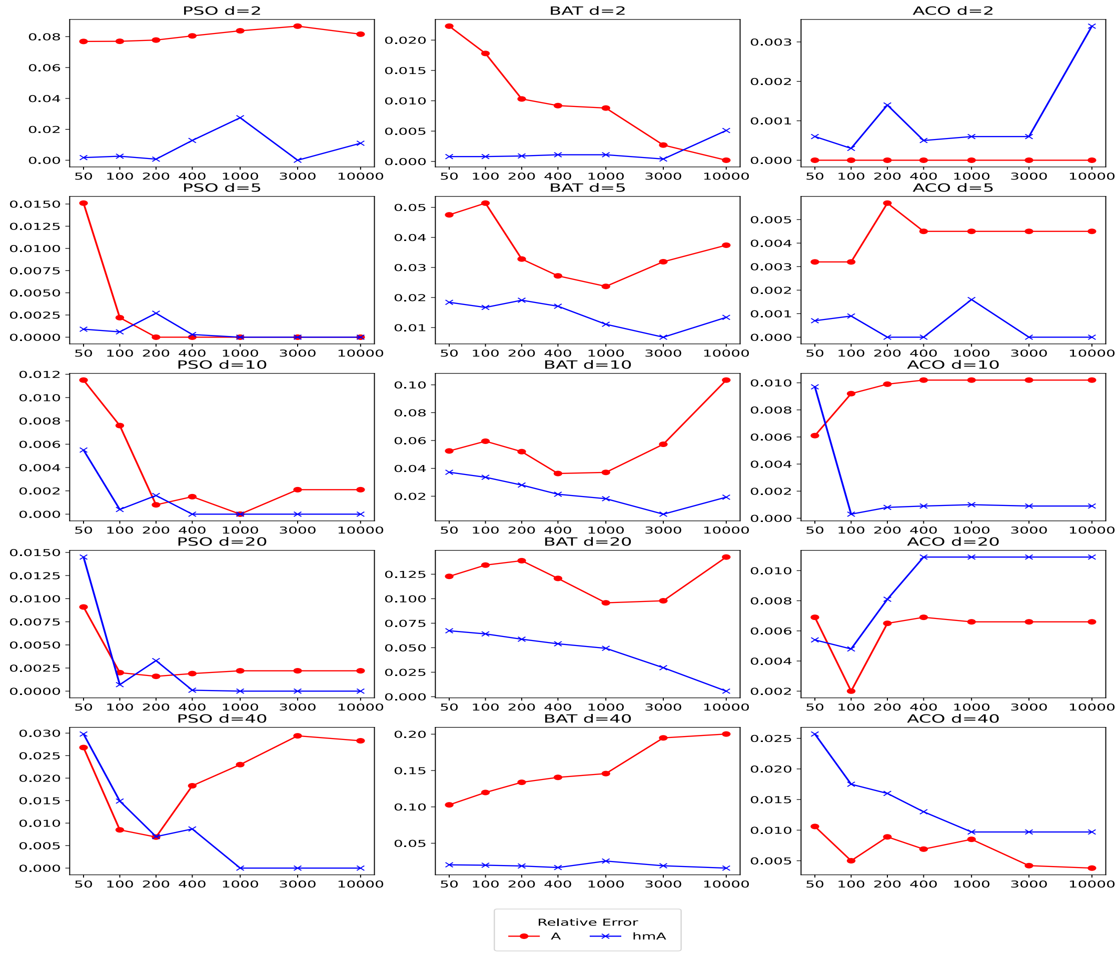

Supplementary Figure 2. Comparative performances of the 3 algorithms displayed in the columns for the first 10000 iterations and summarized by test functions grouped by their dimensions in each row. Each subfigure displays the relative error of (i) A relative to A and hmA combined (red curves), and (ii) hmA relative to A and hmA combined (black curve), where $A = PSO, BAT$ or ACO. The rows show summarized results of 10,000 iterations using test functions grouped by their dimensions.

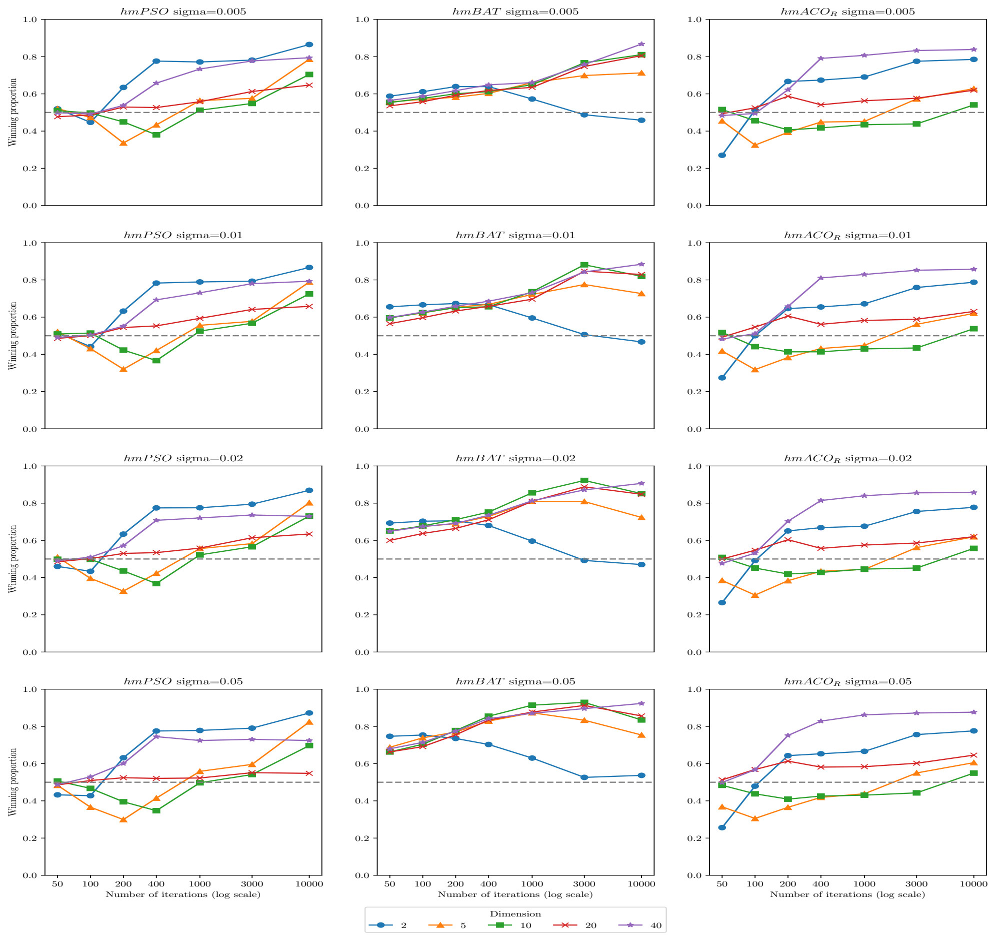

Supplementary Figure 3. Plots of winning proportion of *hmA* against *A* where A = PSO, BAT and ACO categorized according to the dimension of the test functions. Each row shows the result for one value of sigma σ, where $\sigma = 0.005, 0.01, 0.02, 0.05$.

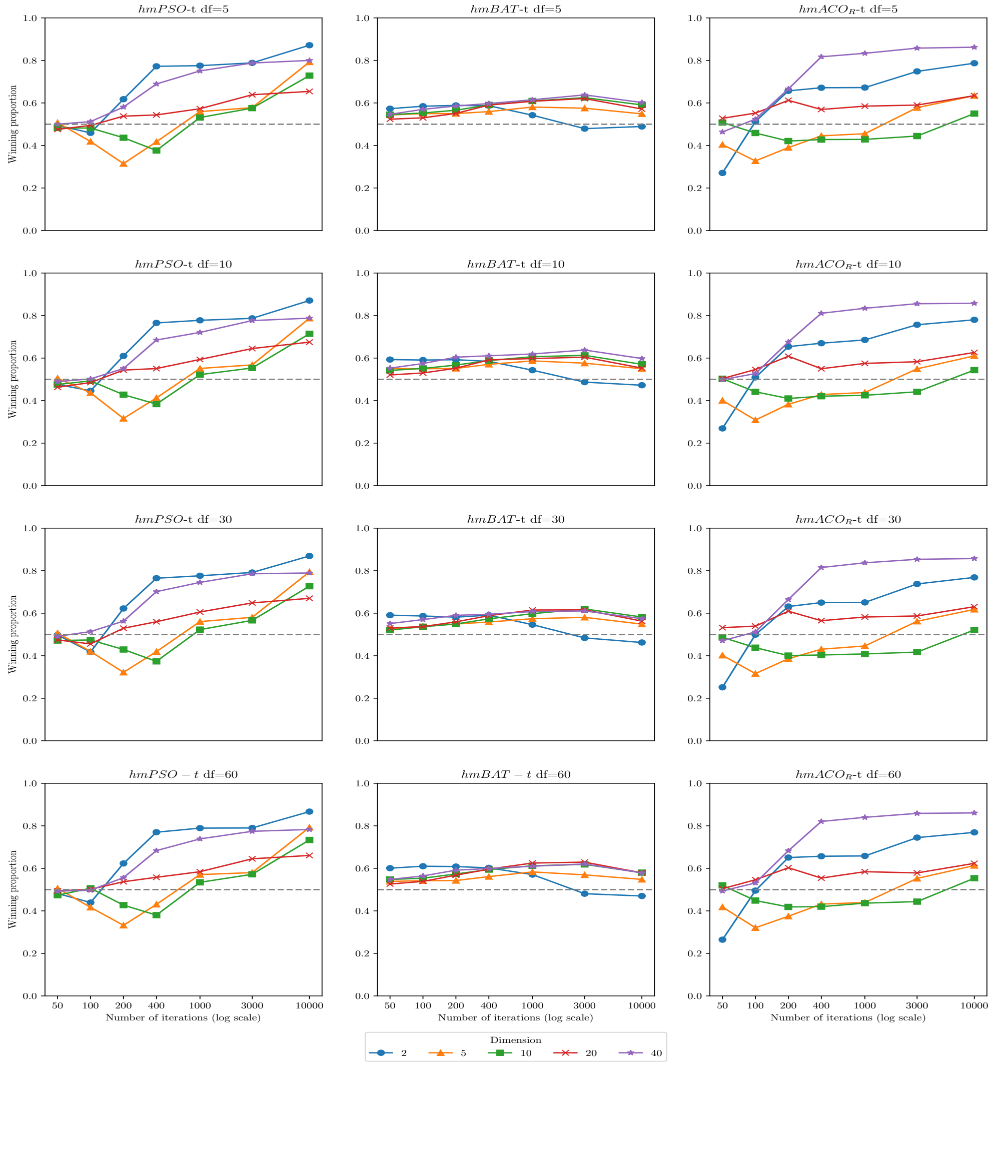

Supplementary Figure 4. Plots of winning proportion of *hmA* against *A* where $A =$ PSO, BAT and ACO categorized according to the dimension of the test functions. Each row shows the result for one value of degrees of freedom df, where $df = 0.005, 0.01, 0.02, 0.05$.

			C01		C02		C04		C05		C20	
			PSO	hmPSO	PSO	hmPSO	PSO	hmPSO	PSO	hmPSO	PSO	hmPSO
d=10	5000	Best	0	0	0	0	16.914	**15.927**	**0**	0.026	**0.35**	0.484
		Median	0	0	261.215	**0**	40.293	**36.829**	3.987	**1.133**	**0.856**	0.883
		Worst	6666.667	**16.597**	4082.274	**0**	**86.238**	106.736	41 357.3	**77.379**	**1.212**	1.533
		Mean	333.573	**0.217**	616.575	**0**	41.834	**38.268**	2801.126	**3.463**	**0.856**	0.895
		StDev	1339.992	**1.667**	916.678	**0**	**13.419**	16.571	6814.004	**10.453**	0.183	**0.182**
		Wins	4	**8**	0	**53**	45	**55**	48	**52**	**54**	46
	10000	Best	0	0	0	0	16.914	**13.708**	**0**	0.026	**0.35**	0.484
		Median	0	0	261.215	**0**	40.293	**35.771**	3.987	**0.64**	**0.84**	0.875
		Worst	6666.667	**16.59**	4082.274	**0**	**86.238**	106.633	41 357.3	**67.961**	**1.212**	1.533
		Mean	333.572	**0.217**	616.575	**0**	41.834	**36.511**	2801.123	**2.194**	**0.834**	0.882
		StDev	1339.992	**1.666**	916.678	**0**	**13.419**	16.601	6814.005	**6.878**	0.182	**0.181**
		Wins	4	**7**	0	**53**	38	**62**	48	**52**	**54**	46
d=30	5000	Best	**0**	0.008	**0**	0.007	122.379	**115.636**	1533.826	**3.248**	2.179	**2.166**
		Median	6666.667	**0.014**	7561.095	**0.014**	247.261	**226.82**	203 444.6	**73.654**	3.302	**3.097**
		Worst	35 000	**0.026**	27 593.83	**0.027**	654.956	**491.749**	1 085 861	**181.685**	5.311	**4.895**
		Mean	9750.031	**0.014**	8515.546	**0.015**	270.636	**235.325**	253 987.9	**60.555**	3.333	**3.174**
		StDev	8403.345	**0.003**	5275.603	**0.004**	93.503	**75.687**	219 015	**42.131**	0.557	**0.516**
		Wins	18	**82**	2	**98**	35	**65**	0	**100**	42	**58**
	10000	Best	**0**	0.004	**0**	0.004	122.379	**88.273**	1533.719	**2.982**	2.177	**2.158**
		Median	6666.667	**0.008**	7561.095	**0.008**	247.261	**212.177**	203 444.6	**71.19**	3.288	**3.024**
		Worst	35 000	**0.012**	27 593.83	**0.012**	654.956	**487.618**	1 085 861	**179.622**	5.167	**4.894**
		Mean	9750.031	**0.008**	8515.546	**0.008**	270.636	**217.677**	253 987.9	**55.655**	3.282	**3.122**
		StDev	8403.345	**0.001**	5275.603	**0.001**	93.503	**78.103**	219 015	**38.628**	0.557	**0.522**
		Wins	18	**82**	2	**98**	28	**72**	0	**100**	46	**54**

			C01		C02		C04		C05		C20	
			PSO	hmPSO	PSO	hmPSO	PSO	hmPSO	PSO	hmPSO	PSO	hmPSO
d=50	5000	Best	1.256	**0.797**	1642.824	**1.421**	371.809	**250.521**	129 948.5	**8.233**	4.305	**4.203**
		Median	20 001.5	**3.573**	20 146.69	**3.941**	593.338	**479.222**	642 337.8	**102.904**	5.864	**5.437**
		Worst	60 000.34	**26.419**	43 517.48	**17.016**	1177.58	**1015.901**	2 339 806	**313.462**	8.39	**8.064**
		Mean	20 652.25	**5.114**	20 254.36	**4.826**	614.594	**505.146**	804 689	**124.569**	5.926	**5.627**
		StDev	10 226.48	**4.374**	9633.153	**3.105**	153.887	**146.273**	495 163.9	**58.418**	0.81	**0.785**
		Wins	2	**98**	0	**100**	24	**76**	0	**100**	38	**62**
	10000	Best	**0**	0.12	1642.503	**0.131**	371.809	**223.52**	129 947.9	**5.087**	4.286	**4.124**
		Median	20 000	**0.22**	20 146.58	**0.234**	593.338	**439.212**	642 335.9	**101.292**	5.846	**5.388**
		Worst	60 000	**0.397**	43 517.47	**0.377**	1177.58	**885.859**	2 339 806	**284.861**	8.39	**7.977**
		Mean	20 600.12	**0.227**	20 254.1	**0.234**	614.594	**467.823**	804 688.9	**119.569**	5.86	**5.525**
		StDev	10 218.19	**0.051**	9633.172	**0.051**	153.887	**143.51**	495 163.9	**54.744**	0.838	**0.787**
		Wins	3	**97**	0	**100**	19	**81**	0	**100**	37	**63**
d=100	5000	Best	33 504.35	**6059.777**	19 232.14	**5329.982**	1101.922	**835.392**	659 695.5	**85.825**	10.247	**8.593**
		Median	64 916.8	**19 146.8**	49 185.95	**12 755.91**	1850.697	**1406.09**	3 249 715	**287.546**	12.913	**12.521**
		Worst	122 117	**49 225.22**	98 765.93	**24 549.34**	3258.137	**2211.948**	7 508 456	**740.376**	22.216	**16.027**
		Mean	67 193.19	**20 225.59**	49 819.8	**13 158.96**	1855.911	**1417.879**	3 367 284	**302.272**	13.412	**12.415**
		StDev	18 544.74	**8172.918**	15 813.37	**3689.659**	342.525	**290.549**	1 539 128	**107.548**	2.239	**1.331**
		Wins	0	**100**	0	**100**	18	**82**	0	**100**	39	**61**
	10000	Best	27 063.87	**405.937**	9585.514	**413.147**	1101.922	**815.556**	659 693.1	**84.314**	9.634	**8.433**
		Median	58 588.46	**1023.273**	44 468.38	**864.394**	1850.697	**1291.438**	3 249 715	**260.456**	12.258	**11.995**
		Worst	116 938.8	**2772.907**	97 726.49	**2353.84**	3258.137	**2084.577**	7 508 456	**680.873**	22.215	**15.86**
		Mean	59 581.93	**1087.452**	45 246.63	**920.817**	1855.68	**1317.909**	3 367 251	**272.238**	13.01	**12.037**
		StDev	19 256.24	**430.677**	16 713.36	**309.62**	342.877	**298.78**	1 539 121	**89.122**	2.324	**1.297**
		Wins	0	**100**	0	**100**	13	**87**	0	**100**	37	**63**

Supplementary Table 3. Comparing the performance of PSO and hmPSO on the five benchmark functions listed in Table 2. Boldface entries denote which algorithm, PSO or hmPSO, outperforms the other. Rows labeled as “Wins” denote the number of times an alogorithm, in 100 runs, beats the other after rounding the values to three decimal places. Ties, when occurred, are not counted.

			C01		C02		C04		C05		C20	
			BAT	hmBAT	BAT	hmBAT	BAT	hmBAT	BAT	hmBAT	BAT	hmBAT
d=10	5000	Best	5169.832	**2849.688**	7830.986	**4214.71**	94.52	94.52	5.697	**0.176**	0.525	0.525
		Median	18 608.97	**11 175.17**	22 843.1	**15 450.4**	285.047	**275.793**	30 543.98	**2.959**	2.207	**1.771**
		Worst	155 393.9	**83 882.07**	260 309.2	**160 977.8**	534.274	534.274	319 530.2	**2922.053**	4.082	**3.938**
		Mean	22 518.56	**14 081.01**	28 754.41	**18 661.36**	294.234	**292.922**	54 519.28	**41.402**	2.045	**1.791**
		StDev	18 330.72	**10 593.38**	29 915.91	**17 967.56**	**96.644**	97.852	64 368.09	**292.754**	**0.798**	0.821
		Wins	1	**99**	1	**99**	16	**19**	0	**100**	21	**70**
	10000	Best	4018.768	**1029.576**	6041.704	**1898.084**	94.52	94.52	0.087	**0.075**	0.524	0.524
		Median	14 410.85	**6627.276**	19 485.46	**10 202.8**	**274.599**	275.793	245.297	**0.128**	2.109	**1.724**
		Worst	124 267.2	**30 315.69**	217 086.8	**82 816.14**	534.274	534.274	34 967.86	**119.353**	4.062	**3.89**
		Mean	18 396.31	**8303.958**	23 363.78	**11 771.08**	292.68	**290.018**	1696.753	**4.679**	1.985	**1.752**
		StDev	14 645.53	**5595.017**	24 118.83	**9832.141**	96.982	**93.68**	4364.951	**19.131**	**0.796**	0.808
		Wins	0	**100**	2	**98**	17	**20**	2	**98**	19	**63**
d=30	5000	Best	41 866.6	**29 762.12**	88 728.51	**64 419.34**	**772.367**	772.371	218 798.8	**264.098**	2.256	**2.175**
		Median	213 505.9	**125 759.9**	329 748.5	**188 760.4**	1172.006	**1167.535**	2 573 303	**19 140.34**	7.938	**5.168**
		Worst	Inf	Inf	15 237 120	**12 595 152**	1577.927	1577.927	Inf	Inf	11.942	**11.503**
		Mean	Inf	Inf	703 696.3	**466 287.1**	1178.139	**1177.628**	Inf	Inf	7.065	**6.002**
		StDev	NaN	NaN	1 671 802	**1 342 108**	187.488	**187.133**	NaN	NaN	**2.656**	2.956
		Wins	0	**98**	0	**100**	**42**	30	0	**86**	22	**78**
	10000	Best	36 209.5	**20 882.44**	73 501.48	**43 076.44**	**772.366**	772.368	13 903.2	**12.028**	**2.174**	2.175
		Median	167 896.5	**78 724.81**	257 596.4	**124 832.3**	1172.006	**1167.534**	652 101.9	**25.612**	7.3	**4.913**
		Worst	Inf	Inf	14 221 846	**9 683 869**	1577.927	1577.927	Inf	Inf	11.877	**11.355**
		Mean	Inf	Inf	599 337.3	**293 220.4**	1178.137	**1177.627**	Inf	Inf	6.692	**5.873**
		StDev	NaN	NaN	1 544 697	**991 283.6**	187.489	**187.133**	NaN	NaN	**2.825**	2.956
		Wins	0	**98**	0	**100**	**49**	28	0	**86**	28	**72**

			C01		C02		C04		C05		C20	
			BAT	hmBAT	BAT	hmBAT	BAT	hmBAT	BAT	hmBAT	BAT	hmBAT
d=50	5000	Best	160 593.8	**124 707.6**	333 326.3	**181 785.2**	1478.713	**1434.677**	1 658 416	**33 223.87**	5.989	**4.299**
		Median	1 141 388	**648 575.5**	1 233 685	**790 199.5**	**2102.768**	2107.243	9 608 472	**709 472.7**	13.356	**11.506**
		Worst	Inf	Inf	Inf	Inf	2806.622	**2767.818**	Inf	Inf	**18.907**	20.44
		Mean	Inf	Inf	Inf	Inf	2116.127	**2115.719**	Inf	Inf	12.428	**10.83**
		StDev	NaN	NaN	NaN	NaN	274.06	**272.808**	NaN	NaN	**4.153**	5.144
		Wins	0	**84**	0	**85**	**51**	44	0	**60**	26	**74**
	10000	Best	145 452.5	**89 488.04**	300 249.2	**112 938.5**	1478.704	**1434.676**	459 267.5	**43.786**	4.72	**4.128**
		Median	906 590.2	**342 432.7**	1 061 133	**500 653.6**	**2102.767**	2107.24	4 178 391	**217.833**	12.704	**11.131**
		Worst	Inf	Inf	Inf	Inf	2806.62	**2767.818**	Inf	Inf	**18.665**	20.256
		Mean	Inf	Inf	Inf	Inf	2116.123	**2115.715**	Inf	Inf	11.468	**10.483**
		StDev	NaN	NaN	NaN	NaN	274.06	**272.807**	NaN	NaN	**4.586**	5.126
		Wins	0	**84**	0	**85**	**52**	39	0	**60**	32	**68**
d=100	5000	Best	1 109 169	**698 364.1**	1 319 385	**792 402.6**	**3410.506**	3421.844	17 760 302	**2 539 323**	13.485	**10.051**
		Median	Inf	Inf	98 134 255	**75 778 988**	4590.797	**4582.679**	Inf	Inf	29.03	**23.833**
		Worst	Inf	Inf	Inf	Inf	**5526.557**	5535.513	Inf	Inf	42.54	**40.724**
		Mean	Inf	Inf	Inf	Inf	4557.909	**4554.62**	Inf	Inf	26.947	**21.866**
		StDev	NaN	NaN	NaN	NaN	**394.169**	397.279	NaN	NaN	**7.898**	10.608
		Wins	0	**36**	0	**52**	47	**53**	0	**22**	17	**83**
	10000	Best	966 598.7	**464 755.1**	934 055.3	**616 067.8**	**3410.457**	3421.818	9 287 841	**20 121.52**	10.969	**8.985**
		Median	Inf	Inf	89 628 278	**50 153 733**	4590.783	**4582.668**	Inf	Inf	27.218	**22.456**
		Worst	Inf	Inf	Inf	Inf	**5526.549**	5535.506	Inf	Inf	41.119	**40.296**
		Mean	Inf	Inf	Inf	Inf	4557.888	**4554.6**	Inf	Inf	23.699	**21.133**
		StDev	NaN	NaN	NaN	NaN	**394.173**	397.281	NaN	NaN	**9.537**	10.711
		Wins	0	**36**	0	**52**	48	**52**	0	**22**	24	**76**

Supplementary Table 4. Comparing the performance of BAT and hmBAT on the five benchmark functions listed in Table 2. Boldface entries denote which algorithm, BAT or hmBAT, outperforms the other. Rows labeled as “Wins” denote the number of times an alogorithm, in 100 runs, beats the other after rounding the values to three decimal places. Ties, when occurred, are not counted.

			C01		C02		C04		C05		C20	
			ACO_R	$hmACO_R$	ACO_R	$hmACO_R$	ACO_R	$hmACO_R$	ACO_R	$hmACO_R$	ACO_R	$hmACO_R$
d=10	5000	Best	0	0	0	0	34.823	34.823	0	0	0.785	**0.7**
		Median	0	0	0	0	**58.267**	58.268	3.126	**0.004**	1.621	**1.559**
		Worst	**17.885**	19.53	0	0	160.182	**159.759**	72.583	**3.994**	2.168	**2.063**
		Mean	**0.23**	0.246	0	0	65.662	**63.663**	4.143	**0.881**	1.572	**1.539**
		StDev	**1.795**	1.958	0	0	26.738	**21.794**	7.852	**1.659**	0.315	**0.287**
		Wins	1	**7**	**0**	**0**	**57**	42	15	**84**	40	**60**
	10000	Best	0	0	0	0	34.823	34.823	0	0	0.66	**0.57**
		Median	0	0	0	0	**58.266**	58.268	3.126	**0**	1.376	**1.354**
		Worst	**17.864**	19.42	0	0	160.182	**159.759**	72.583	**3.987**	2.121	**2**
		Mean	**0.23**	0.245	0	0	65.662	**63.663**	4.13	**0.877**	1.36	**1.328**
		StDev	**1.793**	1.947	0	0	26.738	**21.794**	7.851	**1.66**	0.338	**0.299**
		Wins	1	**7**	**0**	**0**	**57**	42	13	**85**	41	**59**
d=30	5000	Best	0.409	**0**	0.197	**0**	**161.09**	184.066	13.639	**0**	**2.635**	5.458
		Median	457.962	**0**	582.291	**0**	**354.595**	383.216	127.502	**16.49**	8.699	**8.579**
		Worst	10 566.93	**0**	8694.181	**0**	845.723	**734.874**	11 846.88	**86.257**	9.748	**9.449**
		Mean	1920.031	**0**	1328.492	**0**	**384.715**	394.891	752.659	**27.462**	8.512	**8.492**
		StDev	2661.564	**0**	1749.191	**0**	132.83	**117.424**	2135.229	**28.92**	1.092	**0.689**
		Wins	0	**100**	0	**100**	48	**52**	6	**94**	43	**57**
	10000	Best	0.4	**0**	0.196	**0**	**161.09**	184.066	13.639	**0**	2.635	**2.59**
		Median	447.66	**0**	553.069	**0**	**354.595**	383.216	120.813	**3.999**	8.25	**8.201**
		Worst	10 566.93	**0**	8694.181	**0**	845.723	**734.874**	11 846.88	**74.79**	9.43	**9.064**
		Mean	1906.074	**0**	1310.058	**0**	**384.714**	394.89	752.497	**7.345**	7.845	**7.528**
		StDev	2659.219	**0**	1755.17	**0**	132.83	**117.423**	2135.277	**14.455**	**1.552**	1.766
		Wins	0	**100**	0	**100**	48	**52**	2	**98**	40	**60**

			C01		C02		C04		C05		C20	
			ACO_R	$hmACO_R$	ACO_R	$hmACO_R$	ACO_R	$hmACO_R$	ACO_R	$hmACO_R$	ACO_R	$hmACO_R$
d=50	5000	Best	324.045	**0.038**	757.291	**0.06**	509.769	**419.411**	157.777	**0.521**	**5.852**	9.287
		Median	10 792.99	**0.181**	7987.996	**0.163**	845.823	**840.518**	7441.189	**76.451**	16.79	**16.622**
		Worst	**27 476.28**	Inf	27 923.24	**0.541**	1548.269	**1363.497**	282 827.6	**4663.585**	**18.11**	18.414
		Mean	**11 040**	Inf	9062.01	**0.182**	864.562	**848.667**	29 499.15	**112.846**	**16.325**	16.513
		StDev	**6149.615**	NaN	5346.79	**0.094**	**177.324**	198.496	50 003.75	**461.485**	1.767	**1.083**
		Wins	1	**99**	0	**100**	45	**55**	0	**100**	49	**51**
	10000	Best	75.251	**0**	550.704	**0**	509.769	**419.411**	157.777	**0.042**	**4.699**	5.235
		Median	9684.335	**0.002**	6465.612	**0.002**	845.823	**840.518**	7441.189	**36.27**	**16.125**	16.186
		Worst	**27 469.78**	Inf	22 696.57	**0.02**	1548.269	**1363.497**	282 827.6	**144.409**	17.903	**17.901**
		Mean	**9728.688**	Inf	8155.791	**0.002**	864.561	**848.666**	29 499.15	**45.537**	**15.02**	15.403
		StDev	**5779.168**	NaN	5112.924	**0.003**	**177.325**	198.497	50 003.75	**38.51**	3.348	**2.847**
		Wins	1	**99**	0	**100**	45	**55**	0	**100**	**51**	49
d=100	5000	Best	20 411.26	**2142.015**	37 126.72	**2308.284**	**1639.371**	1701.669	47 554.77	**63.5**	16.636	**13.11**
		Median	71 738.22	**6837.408**	57 340.75	**4884.676**	2473.98	**2443.406**	769 228.3	**244.758**	**38.114**	38.151
		Worst	Inf	Inf	Inf	Inf	**3295.681**	3650.44	Inf	Inf	**39.834**	40.154
		Mean	Inf	Inf	Inf	Inf	2509.736	**2450.38**	Inf	Inf	**37.652**	37.918
		StDev	NaN	NaN	NaN	NaN	336.703	**336.145**	NaN	NaN	**2.665**	2.695
		Wins	6	**88**	3	**97**	40	**60**	10	**70**	49	**51**
	10000	Best	14 502.35	**47.834**	24 474.92	**45.784**	**1639.371**	1701.669	42 070.63	**43.36**	11.862	**10.555**
		Median	57 141.5	**157.323**	48 063.85	**121.286**	2473.108	**2443.406**	687 000.7	**221.73**	**37.43**	37.451
		Worst	Inf	Inf	Inf	Inf	**3295.681**	3650.44	Inf	Inf	**39.118**	39.637
		Mean	Inf	Inf	Inf	Inf	2509.519	**2450.376**	Inf	Inf	**35.747**	35.932
		StDev	NaN	NaN	NaN	NaN	336.785	**336.145**	NaN	NaN	6.113	**5.635**
		Wins	4	**93**	2	**98**	40	**60**	4	**77**	**53**	47

Supplementary Table 5. Comparing the performance of ACO_R and $hmACO_R$ on the five benchmark functions listed in Table 2. Boldface entries denote which algorithm, ACO_R or $hmACO_R$, outperforms the other. Rows labeled as “Wins” denote the number of times an alogorithm, in 100 runs, beats the other after rounding the values to three decimal places. Ties, when occurred, are not counted.
